# The populist hotbed: How political attitudes, resentment, and justice beliefs predict both exposure to and avoidance of specific populist news features in the United States

**DOI:** 10.1371/journal.pone.0258220

**Published:** 2021-10-07

**Authors:** Sebastian Scherr, Dominik Leiner

**Affiliations:** 1 Department of Communication, Texas A&M University, College Station, Texas, United States of America; 2 Department of Media and Communication, LMU Munich, Germany; University of Massachusetts, UNITED STATES

## Abstract

A politics of resentment has shaped a low-dialogue political environment in the United States, feeding into populism, and characterized by perceived distributive injustice, detachment between politicians and “the people”, and political polarization. In this political environment, independent of editorial lines, news can spread based on populist content features and drive the political divide even further. However, we still do not understand well, how the forces of political disconnect as well as potentially unifying elements such as political knowledge and the willingness to connect with the other (political) side predict audience interest in populist news featuring *people-centrism*, *anti-elitism*, *restoring popular sovereignty*, and the *exclusion of others*. To better understand what drives (dis-)interest in populist news features, we combined self-report data from a non-student US sample (*N* = 440) on political attitudes with unobtrusively measured data on their selective exposure to populist news. We analyzed the data using zero-inflated negative binomial regression models, in which we simultaneously modeled selective exposure to and avoidance of populist news. The findings indicate that especially the will to connect with others explained exposure to news about *anti-elitism*, especially among Democrats, while Republicans’ news avoidance seems to be specifically geared toward *people-centrism*. Populist communication features promoting “us” vs. “them” dichotomies seem to not automatically resonate with the views of resentful voters and their motivated reasoning.

## Introduction

Populism is here defined as a polarizing set of ideas that support the antagonist construction of ‘us’, ‘the (good) people’ on the one hand and ‘them’, ‘the (bad) elite’ on the other, and politics being responsible for making sure that the general will of the people is heard [1]. In recent years, populism has become a global phenomenon [2,3] with populist movements having been formed and found support across the globe [4]. There are many reasons for the rise in populism, with at least some agreement that a combination of economic developments fostering societal inequalities (e.g., evoked through the global financial crisis) and societal change to the disadvantage of once-strong societal groups (e.g., the factory worker) deserves our attention [3]. Nourished from such developments, especially in the U.S., the notion of a “politics of resentment” has gained traction in explaining the success of political actors such as Scott Walker (45th Governor of Wisconsin, 2011-2019; member of the Republican Party) and Donald Trump by building on the story of undeserving “others” who are seen as getting more than they deserve, thus threatening the “more-deserving self” that is tied to core societal values [5–7]. Thus, politics of resentment is about “who gets what, who has power, what people are like, and who is to blame” (p. 5), it is at base about the resentment of citizens toward other citizens [7].

The study of populist communication typically focuses on *either* the content *or* attitude effects of the ideological phenomenon, usually independent of political color, and oftentimes focusing on specific aspects of populist communication (e.g., blaming others) and their specific audience effects (e.g., on anti-establishment attitudes) rather than more systematically. One reason behind this is that standardized conceptualizations and measurements of populist communication still remain elusive. As an exception, Blassnig et al. [8] recently suggested a multi-dimensional structure to systematically assess different facets of populist communication along the lines of *people-centrism*, *anti-elitism*, *emphasizing the sovereignty of the people*, and the *exclusion of others*. Scholars have just begun to decipher all the facets of the populist ideology’s success [2,9], especially regarding message-specific boundary conditions for individual susceptibilities to populist communication [3].

Importantly, some elements of populist communication has been shown to resonate with audiences that are open to populism [10,11], affect people’s attitudes and behaviors across countries and media channels [12,13], thus supporting a conceptual link between populist communication and populist attitudes. More specifically, Müller et al. [14] show across four nations that exposure to populist communication can intensify pre-existing populist attitudes, especially in a populism-friendly, anti-elitist, and anti-pluralist environment with socioeconomic gaps between larger societal groups such as in the “politics of resentment” [7] climate in the United States.

Hence, although there is some work unraveling the society-riving, centrifugal forces of populism through which people arguably drift off toward the political extremes, scholars have paid much less attention to factors that help overcome political disagreement, bridge political ditches, and re-initiate political discourse and deliberation, or their simultaneous influences. Among the centripetal forces, generalizing from Eveland et al.’s active listening [15], we particularly spotlight people’s will to connect with others and their desire to learn more about politics even if that might go above and beyond someone’s own preexisting knowledge and beliefs.

In order to advance the research field, we followed Blassnig et al.’s conceptualization of populist communication and created a populist news environment to which we exposed individuals and unobtrusively assessed the degree to which people pay attention to populist communication. We used this populist hotbed to explore among U.S. voters from both political camps which of the four core ideological dimensions of populist news (*people-centrism*, *anti-elitism*, *restoring popular sovereignty*, and the *exclusion of others*) grabbed more or less attention, and as the first of its kind, we simultaneously modelled exposure to and avoidance of populist communication as a function of centrifugal (e.g., resentment) and centripetal influences (e.g., will to connect with others). We therefore contribute to the literature by connecting specific features of populist communication to audience attention (and the lack thereof) as a function of individual predispositions. Our contribution is threefold: first, we systematically assess populist communication instead of showcasing some populist news features, and second, we assess how these different dimensions of populist communication resonate with different audiences across the political aisle, and third, we do not exclusively focus on the negative, democracy-threatening forces of populism, but simultaneously consider factors that can help to bring people back together.

### Populism as a news feature: Ideological expression and audience resonance

Populism has been conceptualized as a thin-centered ideology [16]; however, with room for additional vertical and horizontal divides. Yet at its core, populism includes communication features and narratives offering “common sense” solutions to complex problems. These “common sense” solutions are said to arise from the general will of the people [1,16,17], which is not adequately reflected in politics—a notion that applies to many contexts and is easily adaptable to new ones. Thus, the thin populist communication style expresses closeness to “the people”, where politicians aim to appeal to the people and stress sovereignty and the popular will. However, when combined with anti-elitism/anti-establishment positions (vertical division) or the exclusion of certain groups (horizontal division), populism has been redefined as a thicker concept [18]. Similarly, Hameleers et al. hypothesized that a “populist master frame” would be divisive for the people both vertically (contrasting with e.g., corrupt elites) but also horizontally (contrasting with “others”, i.e., societal out-groups) [10].

Importantly, populism offers a coherent narrative of the political world, unfolding against the background of representative democracy: Political elites are attacked because of their lack of responsiveness to and misrepresentation of their constituencies, paired with selfish behaviors. The “cure” proposed by a populist ideology is to reassign control over political decisions to the people. Yet, populism also overstates the homogeneity of the people and develops a suspicious view of groups who are culturally or otherwise different from the majority population. Therefore, populism, can involve “thick” or “thin” communication styles, and as an ideology, goes above and beyond mere rhetorical window dressing [16,18]. It offers sets of discursive political ideas [19]. For example, the virtuous people are pitted against elites and their will is of paramount importance. A society’s problems are said to be caused by the elites or by others [16,17]. The elites are typically depicted as disconnected from the people and as not attending to the people’s needs. Especially within the view of populism as a (thick) communication style, “others” are blamed if “they” are on the same level as “us” (the people) but are not as good or as virtuous as “us” [8,10,18]. Thus, the populist ideology applies to a wide range of situations and political contexts [17,20].

Importantly, populist communication can also include a performative dimension that requires to also take into account who expresses populist ideas (e.g., a journalist, a politician, someone else). However, the present study focuses on populist news features instead of the populist discourse, which positions the object of our analysis closer to what Mazzoleni coined as “media populism” [21].

Notably, populist news features manifest in different ways leading to different forms of populist communication [2,17]. For example, among parties that have been described as right-wing populist, the feature of “exclusion” dominates (e.g. the Freedom Party of Austria [FPÖ]), whereas supposedly left-wing populist parties (e.g. the SYRIZA Party in Greece) are more often characterized by referring to anti-elitist, anti-establishment features [2]. Thus, populism is adaptable to different contexts, making it hard to pin down and research.

Given the applicability and adaptability of populist message features, populist news features have been conceptualized as resonating with individual political attitudes [8,13]. Audience resonance with populist communication is usually explained as a disposition-content congruency [22] in that media effects are stronger if the content is congruent with audience dispositions, including their political viewpoints. Therefore, some authors for example, refer to social identification [23] and self-serving biases to explain in more detail how specific populist messages about the creation, blaming, and exclusion of others (as part of an out-group) would resonate with populist attitudes [13]. Similarly, there seems to be an individual susceptibility to specific aspects of populist communication (e.g., a divide between the people and a corrupt elite and a redistribution of power within a democratic political system) that resonates with individual dispositions that typically include negative attitudes toward the political establishment and political resentment [12]. This was explained by an “insecurity hypothesis,” suggesting that those more strongly affected by societal shifts toward a postindustrial society and by mass immigration would fear a loss of status.

Cramer combined these two lines of argumentation and tied them to the so-called rural consciousness in the United States [7]. She argues that group consciousness creates a shared social identity (rural vs. urban people) with strong in-group perceptions paired with resentment against politics and elite politicians. Cramer [7] repeatedly refers to the group members’ perceptions of not getting their fair share (i.e., procedural, and distributive injustice), and those individual dispositions not only matter for political preferences, but also regarding whether people would engage politically and (re-)connect with others through bipartisanship. Moreover, just world beliefs are plausibly associated with age, since especially older people fear that their communities could fall apart, and that their way of life is threatened by economic inequalities [7].

But how do populism and the politics of resentment resonate with exposure to specific populist news features? Existing evidence suggests that citizens with populist attitudes seem to prefer less traditional, and less politically balanced digital news sources in general [24], and that specific dimensions of their populist attitudes seem to particularly resonate with corresponding populist news features [10], speaking for an increased interest in populist news. At the same time, issue-specific news avoidance can be a result of issue disengagement, which occurs when users are overexposed to specific aspects of an ongoing issue in the news [25].

Specifically, individuals with strong political resentment and crippled justice beliefs might be fed up with reading more about populist news featuring political elites, power imbalances, and distributional inequalities leading to less populist news exposure. From a slightly different angle, one could also argue that one way to bipartisanly re-connect can be through listening to the needs and concerns of others, which could also plausibly be delivered through news media. In combination with the previous argument, while resentment and perceived injustice seem to operate in politically divisive ways (and are possibly associated with less general interest in political news), a willingness to listen, re-connect, and better understand what is going on for the other side could operate in more unifying ways [15]. In fact, due to their simplistic and less deliberative character, populist news might be perceived as attractive for a certain group of people and could therefore be regarded as a vehicle to help connecting with them. Finally, evidence for the unifying potential of political knowledge and its ability to connect and reduce political polarization remains unclear [26]. Having more political knowledge does not automatically imply to also have more balanced information, it can also be related to having more biased information about politics; political knowledge can also come with stronger political opinions especially when the knowledgeable have both more counterarguments for various views on issues and can easily spot argumentative weaknesses in information. However, things might be different for *a desire to have more political knowledge*, which might as well have opposite implications for political information seeking thus motivating the less knowledgeable to expose themselves to a variety of viewpoints to learn. Taken together, we formulate the following hypotheses:

H1: A desire for political knowledge will be associated with an overall stronger exposure to populist news.
H2: The will to connect with other people will be associated with an overall stronger exposure to populist news.
H3: Political resentment and just world beliefs will be associated with an overall stronger avoidance of populist news.

### Attention to specific populist content features in the news

The analysis of how populism is represented in the news media within different political systems has been the focus of considerable research activity in recent years [see e.g., 2]. However, systematic analyses of populist media messages are scarce. One such systematization that has received significant attention [8] suggests that populist communication content in the news media can be best understood along the lines of four dimensions that tie in with the populist communication styles as defined by Jagers and Walgrave [18]. Two dimensions are tied to thin populist communication; namely, *people-centrism* and *popular sovereignty*. The other two dimensions relate to the thick populist communication style: *anti-elitism* and the *exclusion of others*. Importantly, populist media messages vary widely in their content [27], including right-wing populism involving anti-immigrant, racial othering [28], or more abstract differentiations between those who are viewed as deserving more and those seen as deserving less (e.g., economically; having a say in politics); thus, resulting in a climate of popular discontent and disaffection [7].

Politics of resentment [7] shows remarkable similarities to populist communication and can, moreover, be tied to the four dimensions proposed by Blassnig et al. [8]. Firstly, the “good” people are central to the argumentation, and they deserve to have their say (*people-centrism* and *popular sovereignty*). Secondly, there are the evil “others” who get what they do not deserve and, as such, are depriving the good people and should thus be expulsed (*exclusion of others* and *anti-elitism*). Lastly, there seems to be a nostalgic longing for the old (nationalistic) “heartland”, where everything was better [5]. This relates to the populistic notion of a homogenous people in their own heartland [16]. Within the politics of resentment, people feel that the world is unjust to them and that others are to blame for this imbalance [7]. This feeling of injustice then spirals into resentment, with the potential of fragmenting society.

Moreover, those with stronger populist attitudes do not necessarily avoid news more, but seem to use media sources differently from those with weaker populist attitudes [29]. Specifically, they consumed more commercial television and tabloid newspapers, which are often more dramatic, focus more on populist issues [30], and not only follow a populist media logic [31], but also seem to be the preferred news source for those with stronger populist attitudes [10]. Selective exposure to populist news is therefore likely to occur differently among citizens. Given a lack of content-specific research on the selective exposure (or avoidance) of specific populist news features, we formulated two research questions that more specifically differentiate between the four content dimensions of populist communication [8]:

RQ1: To what degree is a desire for political knowledge and a will to connect with others associated with exposure to specific features of populism (i.e., *anti-elitism*, *restoring sovereignty*, *people-centrism*, *exclusion of others*)?
RQ2: To what degree are resentment and just world beliefs associated with avoidance of specific features of populism (i.e., *anti-elitism*, *restoring sovereignty*, *people-centrism*, *exclusion of others*)?

## Method

### Participants and power

A total of *N* = 440 individuals (222 women, 217 men, and one participant indicating “other”) were included in this study. Participants were on average *M*_*age*_ = 41 years old (*SD*_*age*_ = 12.55; range: 18–72 years) and 71.5% had an associate or college degree. The resulting sample was mostly identified as Caucasian (83.6%), but also included African Americans (8.6%), Asians (6.8%), Hispanics or Latinos (6.4%), and Native Americans (1.8%). Three individuals did not respond to the question regarding racial identification. Thus, compared to the 2015 US census data, our sample overrepresents Caucasians (77%), Asians (5%), as well as Native Americans and Pacific Islanders (1.4%), while Hispanics (17%) and African Americans (13%) are underrepresented. With regard to their political orientation, on a scale ranging from 0 (*extreme political left*) to 10 (*extreme political right*), the participants answered on average *M* = 4.51 (*SD* = 2.83; range: 0–10; *Mdn*: 5.00). Regarding their political participation, 47.7% indicated that they had voted in all four previous elections, while only 7.3% reported not having voted in any of them (participation in Presidential Election 2012: 73.2% vs. Presidential Election 2016: 82.7%; Mid-Terms 2014: 51.6% vs. Mid-Terms 2018: 78%). In the last election (i.e., Mid-Terms 2018), 54.8% of the participants voted for the Democratic Party (38.5% for the Republican Party), and 24.8% furthermore indicated having voted differently in some of the three previous elections (16.2% among Democrat voters; 31.1% among Republican voters).

Based on an *a priori* power analysis using G*Power 3.1.9.2—assuming only small effects (*ΔR*^*2*^ = .02) and explained variances *R*^*2*^ ranging between .08 and .49 [thus residual variances ranging between .92 and .51] [see e.g.,^32^]—, the required sample size would need to be between *N* = 151 and 202 in order to detect small effects of ρ^*2*^ = .02 with a statistical power between .80 and .90 in a two-tailed test. Thus, separate group analyses for self-reported Democrats (*N* = 235) and Republicans (*N* = 165) should have achieved a statistical power of .94 and .83 respectively based off of a post-hoc power analysis.

### Procedure

In November 2018, shortly after the 2018 US Mid-Term Elections, Amazon Mechanical Turk (MTurk) users over 18 were invited through the platform to participate in a study on political news in America if they were US citizens and had an MTurk approval rate of at least 95% [see 33]. A reimbursement for study participation was granted through the platform. The study was approved by the university’s Ethical Review Board (Dossier No. G-2018 11 1420). After giving their informed consent, the participants first answered demographic questions and a series of questions related to the study’s topic of American politics and news media. After completing the first part of the survey, a link to a news web magazine was displayed to the participants [34]. The magazine was introduced as a new online news portal that collects news articles on one topic—in this case, politics. The participants were informed that they could read for up to 5 minutes on the website as they would normally do, before they were to be redirected to the second part of the survey. It was clearly stated that the available reading time would not be enough to read all the articles in detail and that they would have to choose. The participants could also interrupt this process and proceed with the survey earlier if they wanted to. Upon clicking the forwarding link, a pop-up window opened, and participants automatically landed on the web magazine. After the reading time was up, the participants received a browser warning that their reading time was ending, the pop-up window closed, and the participants were automatically forwarded to the second half of the survey.

The answers from both parts of the survey together with the digital traces of individual browsing patterns were paired up and merged, allowing for us to relate their information-seeking behavior with their individual characteristics. Finally, 13 participants who failed to correctly answer all four attention checks (e.g., “click on *strongly disagree*”) placed throughout both surveys, who indicated that they had “often just clicked somewhere to get it done quickly,” or who had been “distracted by [the] environment (people, noises, etc.) SEVERAL times” were excluded from the data analysis resulting in the final sample (*N* = 440).

### Measures

#### Selective exposure to populist news features

Selective exposure was measured as the time in seconds spent on the web magazine up to a maximum of 5 minutes so that the participants had to make choices. The web magazine consisted of eight news articles and an overview landing page. Digital trace data from the web magazine were unobtrusively logged by software. As such, the exact viewing time for each news article was monitored in seconds. The participants were free to choose what to read.

**Fig 1:**
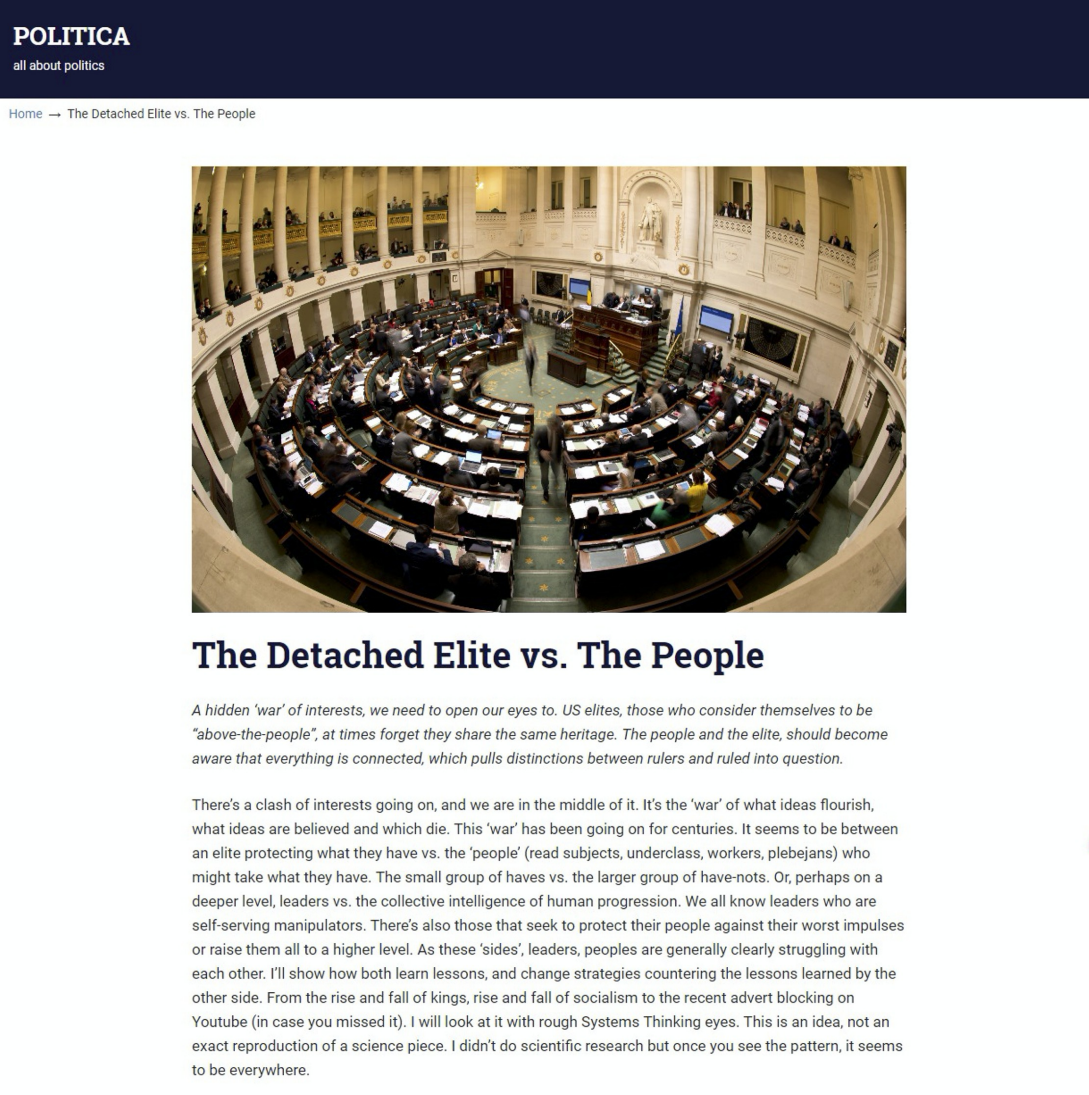
Screenshot of the landing page of the news magazine.

**Fig 2:**
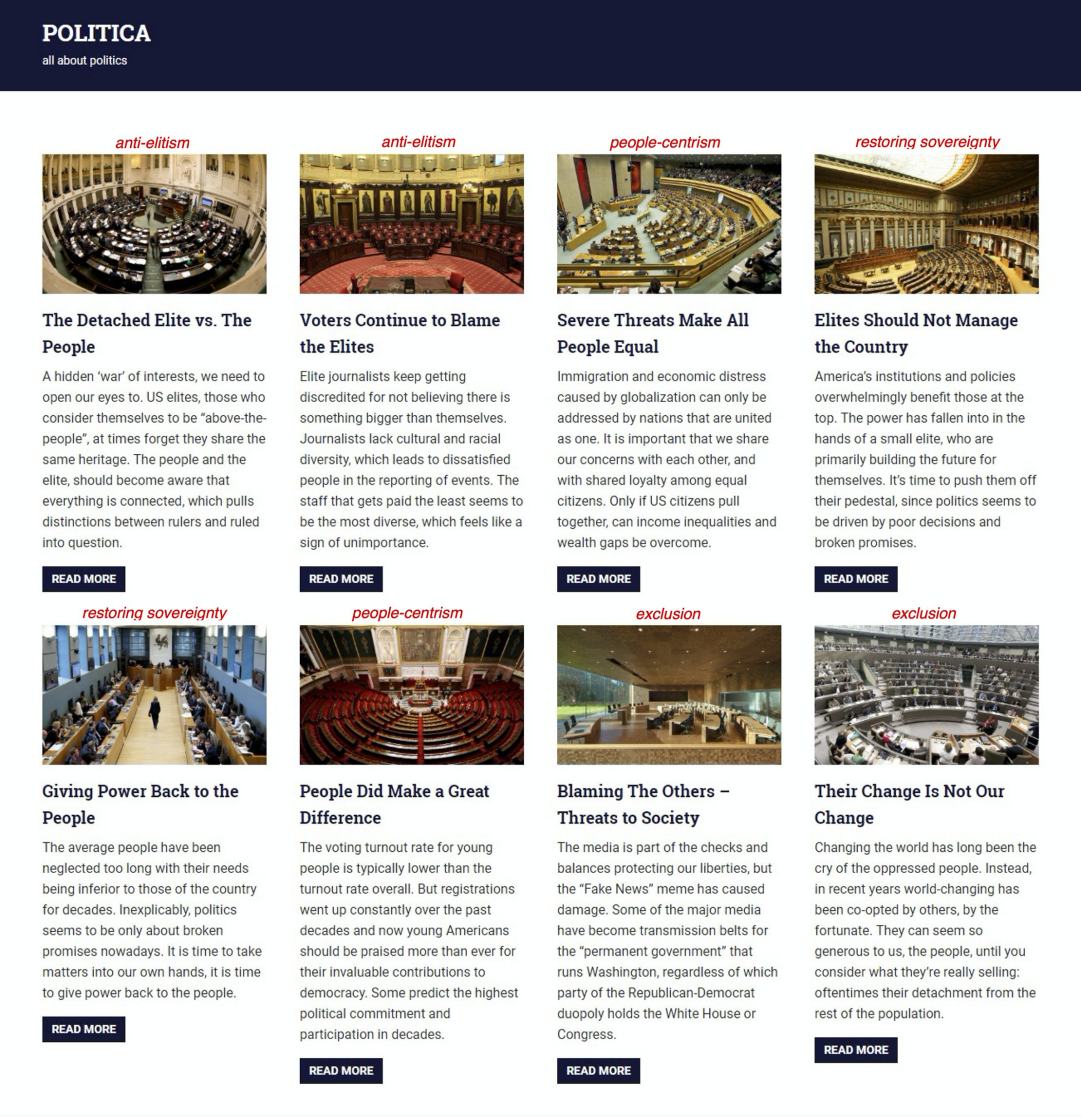
Screenshot of an example article reflecting the “anti-elitism” dimension.

The news website contained eight news articles taken from different, quality news media outlets in order to reflect the tone of populist news topics at that time. However, the articles were edited in order to be of comparable length. All eight articles were presented at the same time, but in a randomized order on the website to rule out any presentation order effects. Pictures were held constant in order to rule out possible visual effects on news selection. Following stimulus sampling [35] in order to reduce the likelihood of a single stimulus’ unique characteristics impact the results, we used two different articles representative of each of the four dimensions suggested by Blassnig et al.’s [8] conceptual model of populist communication (i.e., *anti-elitism*, *restoring sovereignty*, *people-centrism*, and the *exclusion of others*) resulting in a total of eight articles on the web magazine’s landing page (see Fig 1). The stories covered political topics, but they varied widely, including, for example, topics on young voter turnout, fake news, immigration, and globalization. Each article on the landing page of the web magazine was represented as a headline (six words), a brief teaser (50 words), and a picture of a parliament (see Fig 1). After clicking on one of the pictures, headlines, or teaser texts, the participants were forwarded to the full article (see Fig 2), which was exactly 800 words long in every case in order to keep the formal aspects as constant as possible. The participants could move back and forth between full articles and the landing page (overview) as they pleased. All the articles had been pretested with an independent MTurk sample (*N* = 35, 42.9% female; *M*_*age*_ = 41.0, *SD* = 10.6; no enrolled students; 91.4% Caucasian, 5.7% with a Hispanic or Latino background) to ensure that all the headlines were perceived in line with the intended populist dimension.

Within the time window of up to 5 minutes, individuals could choose to either read a populist news article (unobtrusively monitored in seconds), which automatically meant that they did not read all other articles at that point in time. We then summed up the time (in seconds) individuals spent on each one of the eight articles, and if an article had never been chosen, it was coded as zero (i.e., avoided). *Overall exposure to populist news features* was then operationalized as the sum of all reading times collected across all eight news articles and *overall avoidance of populist news features* reflected all the coded zeros for non-exposure. Since there were always two articles representing the same populist features, we also summed up the times for those in order to reflect the exposure to specific populist features or coded a zero when a feature was avoided.

#### Desire for political knowledge

Two items were used to assess, with sufficient internal consistency (2 items; Cronbach’s *α* = .790; *r* = .656, *p* < .001; *M* = 2.50, *SD =* 1.30), whether there was an intrinsic desire to know more about politics (i.e., “Even if it upsets me, I want to know more about politics” and “It is important to know a lot about politics”). The agreement with both statements was assessed on a 7-point Likert-type scale ranging from 1 = *strongly disagree* to 7 = *strongly agree*.

#### Will to connect with others

Furthermore, three statements captured whether reading news functioned to (re-)connect with other people, independent of their political views (e.g., “Reading about politics provided me with material for a discussion with people holding a different viewpoint,” “Reading about politics provided me with material to talk about with like-minded others,” and “Reading about politics provided me with useful information to better understand people holding a different viewpoint”). All the statements were assessed on a 7-point scale ranging from 1 = *strongly disagree* to 7 = *strongly agree*, to which participants answered sufficiently consistently (3 items; Cronbach’s *α* = .882; *M* = 4.84, *SD =* 1.44) so that we could then transform these statements into one composite measure.

#### Resentment

We captured resentment as a composite measure of four statements about the individual’s characteristic feelings of frustration (e.g., “I am sometimes eaten up with jealousy”) and bitterness (e.g., “I wonder why sometimes I feel so bitter about things”) about things in life [36]. All the statements were assessed on a 7-point scale (1 = *extremely uncharacteristic of me* and 7 = *extremely characteristic of me*) and yielded in a sufficiently consistent composite measure (4 items; Cronbach’s *α* = .853; *M* = 3.24, *SD =* 1.60).

#### Just world beliefs

Just world beliefs were judged on 7-point scales (1 = *strongly disagree* and 7 = *strongly agree*) and describe a four-dimensional construct (procedural vs. distributive justice; self vs. others) [37]. Each of the four dimensions is captured with four statements: *procedural justice* reflects either one’s own (e.g., “People usually use fair procedures in dealing with me”) or other people’s experiences of fair decision processes, rules, and treatments (e.g., “Other people usually use fair procedures in dealing with others”). *Distributive justice*, on the other hand, includes statements about oneself (e.g., “I usually get the outcomes that I deserve”) or others (e.g., “Other people usually get the outcomes that they deserve”). However, principal axis factoring with Promax (κ = 4) rotation showed that the items sort along two main factors (KMO = .948; Bartlett’s test of sphericity: χ^2^ (120) = 8456.70, *p* < .001), yielding a two-factor structure among all statements that explained 73.39% of the observed variance. We therefore created two composite measures for *procedural* (8 items; Cronbach’s *α =* .952; *M* = 4.63, *SD* = 1.22) and *distributive justice beliefs* (8 items; Cronbach’s *α =* .957; *M* = 4.58, *SD* = 1.33).

#### Covariates

We included a dummy variable for gender (50.5% female) and education (72% with a college degree), together with age and the self-reported party they voted for in the 2018 US Mid-Term elections (54.8% Democratic Party) in the regression models.

### Data analysis

A zero-inflated negative binomial regression model seemed most appropriate to model the selective exposure data for three reasons: First, the collected digital trace data for information seeking inherently produced excessive zeros stemming from the other articles that a participant did not choose to read at a given time. Second, the data were over-dispersed, meaning the variance for the outcomes is quite large relative to their means, and third, monitored reading times (in seconds) are non-negative, integer values that can be treated as count data. This type of regression basically assumes that all possible observations are based on two different processes: People have read certain news articles, or they have not, and if they have not, the only possible outcome is zero. If they have read articles on the news website, each participant is likely to have spent a unique amount of time on the website that is reflective of a count process. A zero-inflated negative binomial regression then estimates not reading on the website (i.e., the excess zeros) as a logit model with a unique set of predictors and combines this with a different set of predictors for the count process of reading the news (i.e., the non-zero observations) as part of a negative binomial model. In other words, rather than using the same set of variables to predict selective exposure to populist news features within one model, a combination of two different sets of predictors is used: The first set predicts news exposure (predictors: gender, age, education, political orientation, desire for political knowledge, will to connect with others [and interaction terms]), and the second set predicts news avoidance (predictors: resentment, procedural justice beliefs, distributive justice beliefs [and interaction terms]). Consequently, as depicted in Table 1, the predictors within each set were moderately correlated with one another, while the correlations between the two sets of predictors were low or absent.

**Table 1:** Zero-order correlations between predictors of selective exposure to populist news.

		1	2	3	4	5	6	7	8	9
1	(RT) Anti-Elitism	—								
2	(RT) Restoring Sovereignty	.257	—							
3	(RT) People-Centrism	.028	.159	—						
4	(RT) Exclusion of Others	-.059	-.138	-.012	—					
5	Desire for Political Knowledge	.126	-.148	.015	-.009	—				
6	Will to Connect with Others	-.088	.236	-.019	.117	-.464***	—			
7	Resentment	-.056	.000	-.041	-.032	.084	.090	—		
8	Procedural Justice	.070	.025	.141	.050	-.077	.099*	-.256***	—	
9	Distributive Justice	.013	.057	.019	.007	-.040	.093	-.261***	.756***	—
	*M*	88.2	90.4	100.8	102.5	2.50	4.84	3.24	4.63	4.58
	*SD*	71.6	67.2	74.8	72.2	1.30	1.44	1.60	1.22	1.33

*Note*. *N* = 440; Pearson correlations with two-tailed significance tests.

RT = Reading time in seconds; desire for political knowledge and the will to connect with others, and just world beliefs were all assessed on a 7-point scale ranging from 1 = *strongly disagree* to 7 = *strongly agree*; resentment was assessed on a 7-point scale ranging from 1 = *extremely uncharacteristic of me* to 7 = *extremely characteristic of me*.

* *p* < .05

** *p* < .01

*** *p* < .001.

## Results

Overall, *N* = 440 participants spent on average 1.45 minutes on the website (*M* = 86.71 s, *SD* = 101.76 s; *Mdn* = 32; range: 0–295 s) and read on average *M* = 1.08 (*SD* = 1.30) articles. Participants spent more time on articles focusing on the *exclusion of others* (*M* = 102.54 s, *SD* = 72.17 s; *n* = 104) and *people-centrism* (*M* = 100.84 s, *SD* = 74.79 s; *n* = 95) than on articles about *restoring sovereignty* (*M* = 90.39 s, *SD* = 67.18 s; *n* = 118) and *anti-elitism* (*M* = 88.21 s, *SD* = 71.59 s; *n* = 82). Due to zero inflation and the non-normal distribution of the reading times, it comes as no surprise that the zero-order correlations between reading times and the predictor variables are only small (see Table 1).

Against this backdrop, addressing H1-H3, our analyses show that neither a desire for political knowledge (*B* = −.040, *SE* = .056, *p* = .470) nor a will to connect with others (*B* = .003, *SE* = .049, *p* = .953) were associated with the overall exposure to populist news (see left column in Table 2). A simultaneously estimated logit model explaining overall avoidance of populist news shows that neither procedural justice beliefs (*B* = -.113, *SE* = .197, *p* = .566), nor distributive justice beliefs (*B* = .209, *SE* = .196, *p* = .288) helped explaining populist news avoidance, but resentment did (*B* = .271, *SE* = .090, *p* = .003). We therefore reject H1 and H2, and only partially accept H3, specifically for resentment, which seems to be associated with avoiding populist news overall.

**Table 2:** Zero-inflated negative binomial regression model simultaneously predicting selective exposure to and selective avoidance of populist news features.

	Overall Exposure			*Anti-Elitism*			*Restoring Sovereignty*			*People-Centrism*			*Exclusion of Others*		
	*B*	*SE*	*p*	*B*	*SE*	*p*	*B*	*SE*	*p*	*B*	*SE*	*p*	*B*	*SE*	*p*
*Negative Binomial Model Explaining Exposure to Populist News Features*															
Gender (female)	.051	.077	.507	**.454**	**.168**	**.007**	.129	.140	.360	-.004	.155	.982	.155	.142	.276
Age	-.005	.003	.161	-.012	.009	.163	-.001	.007	.867	-.007	.005	.196	-.008	.006	.205
Education (college)	-.102	.079	.197	-.100	.187	.594	-.162	.148	.274	-.043	.160	.789	-.149	.149	.317
Political Orientation (Democrat)	.025	.456	.956	**2.506**	**.748**	**.001**	1.163	.942	.217	1.261	.844	.135	.308	1.274	.809
Desire for Political Knowledge	-.040	.056	.470	-.113	.099	.251	-.042	.081	.606	-.178	.195	.360	.129	.200	.519
Will to Connect with Others	.003	.049	.953	**-.303**	**.116**	**.009**	.038	.116	.740	-.138	.083	.096	.067	.152	.660
Pol. Ori. × Desire for Pol. Know.	-.027	.078	.727	-.089	.141	.526	.066	.123	.595	.146	.214	.493	-.028	.217	.896
Pol. Ori. × Will to Connect	.020	.070	.775	**.513**	**.144**	**<.001**	.160	.145	.270	.176	.109	.108	.088	.172	.608
*Logit Model Explaining Avoidance of Populist News Features*															
Resentment	**.271**	**.090**	**.003**	**.323**	**.115**	**.005**	.118	.111	.287	**.266**	**.112**	**.017**	.122	.094	.195
Procedural Justice	-.113	.197	.566	-.140	.291	.631	.184	.218	.397	-.301	.269	.263	-.024	.250	.925
Distributive Justice	.209	.196	.288	.181	.263	.492	-.073	.223	.745	.217	.265	.413	.187	.244	.443
Pol. Ori. × Resentment	-.195	.114	.085	-.242	.158	.126	-.125	.132	.343	**-.340**	**.142**	**.017**	-.036	.122	.765
Pol. Ori. × Proc. Justice	.103	.253	.684	.310	.334	.353	.176	.268	.511	.248	.315	.432	-.023	.292	.937
Pol. Ori. × Distr. Justice	-.028	.248	.912	-.178	.317	.573	-.199	.267	.456	-.097	.309	.753	-.025	.292	.931
*Overall Model Parameter*															
Log Pseudolikelihood	-1594.035			-565.854			-823.311			-681.527			-742.176		
Wald *χ*^2^ (8)	9.07			24.64			13.66			5.18			8.87		
*p*	.337			.002			.091			.738			.354		

*Note*. Total sample size *N* = 440; robust standard errors reported; zero-inflated negative binomial regression model that predicts both the absolute time spent with specific content features of populist communication and their avoidance (i.e., not reading) as two separate functions combined into one regression model. Reading times were unobtrusively monitored, measured and reported in seconds. Each of the four dimensions of populist communication features [8], were represented by two articles in a web magazine. Higher numbers are indicative of higher agreement with the construct described. Overall exposure to populist news features is the sum of all exposures to any of the four dimensions as reflected in the news articles by an individual, while overall avoidance of populist news features is modeled as the non-exposure to all other populist news features that have not been chosen at the same time. The model includes individuals who indicated having voted either for the Democratic Party (*n* = 235) or for the Republican Party (*n* = 165) in the 2018 US Mid-Term Elections.

The model includes dummies for gender (*male* = 0, *female = 1*), education (*less than any college* degree = 0, *any college degree = 1*) and political orientation (*Republican Party Voter* = 0, *Democratic Party voter* = 1); continuous measures for resentment (7-point scale ranging from 1= *extremely uncharacteristic of me* to 7 = *extremely characteristic of me*), a desire for more political knowledge, the will to connect with others, as well as indicators for procedural and distributional injustice (1 = *strongly disagree* and 7 = *strongly agree*).

We then formulated two research questions about the degree to which these variables were helpful in explaining exposure to specific features of populism (see RQ1 and RQ2). Answering RQ1, data analysis shows that a will to connect with others is connected to less exposure to populist news featuring *anti-elitism* (*B* = -.303, *SE* = .116, *p* = .009). However, having voted for the Democratic Party in conjunction with stronger will to connect with others made exposure to news featuring anti-elitism more likely (*B* = .513, *SE* = .114, *p* < .001). A desire for political knowledge on the other hand was not significantly associated with exposure to this or any other of the four populist news features under investigation, which also seems to not be moderated by political orientation (see Table 2). Finally, RQ2 asked for the relevance of resentment and just world beliefs for the avoidance of populist news features. The data shows that resentment helped in explaining the avoidance of anti-elitism features (*B* = .323, *SE* = .115, *p* = .005) and people-centrism (*B* = .266, *SE* = .112, *p* = .017). A significant interaction between resentment and political orientation furthermore shows that having voted for the Republican Party in conjunction with resentment increased the odds of avoiding news featuring people-centrism (*B* = -.340, *SE* = .142, *p* = .017). Procedural and distributive justice beliefs on the other hand did not explain the avoidance of specific populist news features.

Finally, following up on these findings, there were no significant differences between Democrats and Republicans regarding their will to connect with others (*M*_*Dem*_ = 4.97, *SD*_*Dem*_ = 1.36; *M*_*Rep*_ = 4.77, *SD*_*Rep*_ = 1.48; *t*(398) = 1.417, *p* = .157) or their levels of resentment (*M*_*Dem*_ = 3.31, *SD*_*Dem*_ = 1.57; *M*_*Rep*_ = 3.11, *SD*_*Rep*_ = 1.66; *t*(398) = 1.189, *p* = .235), but Democrats had a significantly lower desire for political knowledge than Republicans did (*M*_*Dem*_ = 2.23, *SD*_*Dem*_ = 1.09; *M*_*Rep*_ = 2.62, *SD*_*Rep*_ = 1.29; *t*(398) = 3.258, *p* < .001).

## Discussion

The success of populism [2,4] has raised questions about populist communication in the media [21], and about how populism resonates with voters and supporters [10,11]. However, systematizations of specific populist news features are still scarce [8], and they have not been used to more specifically address which individual political beliefs and attitudes actually predict the selection or avoidance of specific dimensions of populist communication. Existing studies related populist attitudes broadly [38] to either self-reported exposure to populist media content [10], or focused on a single dimension of populist communication features [e.g., blaming others; 11] usually limiting the amount of news choice decisions per participant [11]. Despite these shortcomings, it seems as if at least some attitudinal congruence drives exposure to what Mazzoleni coined as “media populism” [21]. While these findings are of paramount importance to better understanding the success of populism, there have always been observations of participants who chose not to read populist news. For example, in one study it was noted that “those participants who self-selected a control condition [i.e., an article without populist features] have significantly lower populist attitudes than those who self-selected treatment conditions [i.e., containing populist content features]” [11]. Although not reading about populist news is a legitimate choice for media users, this path has not been fully integrated yet, which is why our current understanding of populist news selection is still incomplete.

The present study aimed at contributing to this line of research in at least XX ways: First, we focus on core features of populist news content (*anti-elitism*, *restoring sovereignty*, *people-centrism*, *exclusion of others*) and unobtrusively monitored through a mock news website how individuals chose to read populist news. Second, we used zero-inflated negative binomial regressions to simultaneously model both the exposure to and the avoidance of populist news features using two distinct sets of predictors. These predictors were ingrained in what Cramer coined as “politics of resentment” [7]. This conceptualization typically looks at resentment, feelings of distributive and procedural injustice, a lost connection with the other side of the votership, and a reduced desire to occupy oneself with politics as for why people got politically charged. Third, in doing so, we sought to offer an alternative explanation for why individuals expose themselves to populist messages in the news that goes above and beyond the almost tautological rationale that populist attitudes would resonate more strongly with some audiences and therefore lead to more exposure to media populism.

We would like to focus on the findings for two particular populist news features: *anti-elitism* and *people-centrism*. Based on a sample of US citizens (*N* = 440) and data having been collected in the direct aftermath of the 2018 Mid-Term Elections, analyses show that the will to connect with like-minded people explained overall less exposure to *anti-elitism*, but more exposure among recent Democrat voters. Apparently, the *anti-elitism* dimension of populist communication fulfills a different function for both sides of the US votership. One possible interpretation in the given context of the 2018 US Mid-Term Elections could be that Democrats regarded news against the political establishment as a way to better understand how others (including Republican voters) might think and therefore read more about it. Resentment drove the avoidance of anti-elitist news, which, taken together, could be interpreted as a sign of issue disengagement due to possible overexposure to anti-elitist news in the run-up of the election.

Second, *people-centric* news dealt with how shared loyalty among US citizens would help overcome problems related to immigration and economic stress, how sticking together would prevent people from losing more ground, but it also stressed high voter turnout, alongside political commitment, and participation among young voters. Interestingly, resentment explained the avoidance of people-centric news, particularly among recent Republican voters. One explanation oif this finding could be that resentment is an expression of “othering”, i.e., of being dissatisfied with politics and someone’s own fair share in it. Resentful individuals might therefore not want to read about how citizens have to stick together to overcome political mischief. Apparently, resentment—a central tool for Cramer’s explanation of political polarization—resonates both with the avoidance of *anti-elitist* and *people-centrist news*. Both populist features have in common that they construct an ingroup, an “us,” “the people,” and that might not easily align with prior resentful beliefs toward politics. It might be that “us” vs. “them” dichotomies in the news generally resonate with individuals who accept populist ideas, but they do not necessarily resonate with individuals who are resentful toward politics, whose motivations are more individualistic, and who, thus, cannot align such grouping dichotomies with their own identity as part of their motivated reasoning [39].

Ultimately, our findings support the notion of a more detailed differentiation between specific dimensions of populist communication to understand how these resonate with individual attitudes and prior beliefs. A closer look at this seems especially relevant to identify topics that resonate with procedural and distributive justice beliefs, for which we could not observe significant relationships, and that resonate with both political camps and that can be used to spark a bipartisan dialog. Our findings could be interpreted in a way that resentment is a useful tool through which to start such a dialog (in contrast to procedural or distributive injustice).

### Limitations

The study has important limitations that must be kept in mind. First, in order to predict exposure to specific features of populist news, the participants were placed within a populist hotbed that only contained populist news. Sets of different news articles (including non-populist ones) would be more reflective of a scenario of selective exposure to different news items. Future studies could compare non-populist news items not used alongside populist news items in order to obtain an even more refined picture of selective exposure to populism. Moreover, although we included attention check items as well as items that assessed the diligence and distraction with which individuals answered the survey, there is a possibility that some rushed through in order to simply collect their compensation. A modified study design could leave the entire information seeking task optional and competing with other, less attractive tasks (e.g., counting the number of letters of the ingredients of prescription drugs). This modification could help disentangling rushing from news avoidance. Relatedly, the context was geared toward the American 2018 Mid-Term context, and the cultural differences involved in populism could be better reflected in future studies. Thus, our data reflect an election-time scenario, which differs from non-election times due to the flood of election-centered news. Therefore, it would be interesting to replicate this study during a non-election period. Moreover, although the initial selection of news articles is made based on the article headlines and teaser text, it is possible that the full articles differed in ways unrelated to the main themes we primarily focused on in our study, which might have driven observed effects. Finally, scholars continue to question the validity of MTurk samples, especially due to the demographic differences arising from probability samples. However, methodological research has shown that MTurk may yield comparable results for, for example, political ideology [40], especially when certain additional precautions (e.g., screening questions for problematic responders) are taken [41] and demographic or political background variables are controlled for [42]. However, our data are not comparable to population-based samples and should therefore also be replicated using different samples.

## Conclusion

In the direct aftermath of the 2018 US Mid-Term Elections, we investigated the degree to which two different sets of 1) political attitudes, and 2) resentment and just world beliefs would be predictive of 1) selective exposure to and 2) avoidance of four typical populist news features (*anti-elitism*, *restoring sovereignty*, *people-centrism*, *exclusion of others*). In order to overcome the typical methodological problems of selective exposure research (zero inflation of the data; non-normal distribution of data), we used a zero-inflated negative binomial regression model that estimates the two processes separately using two different functions and then combines them as part of the same statistical model. The findings suggest that a will to connect with other people (both like-minded people and those with cross-cutting political views) helped in explaining exposure to *anti-elitism* among recent Democrat voters, while resentment explained the avoidance of *people-centric news* among Republicans. While populist communication oftentimes creates dichotomies along the lines of “us” vs. “them,” our findings seem to support the notion that resentful individuals share more individualistic motives that do not align with certain populist news features. Motivated reasoning could be a viable explanation for these observations that would deserve further investigation.

## References

[1] Mudde C. The populist zeitgeist. Government and Opposition. 2004;39(4):541–63. doi: 10.1111/j.1477-7053.2004.00135.x

[2] Aalberg T, de Vreese CH. Introduction: Comprehending populist political communication. In: Aalberg T, Esser F, Reinemann C, Strömbäck J, de Vreese CH, editors. Populist political communication in Europe. New York, NY: Routledge; 2017. p. 3–11.

[3] de Vreese CH, Esser F, Aalberg T, Reinemann C, Stanyer J. Populism as an expression of political communication content and style: A new perspective. The International Journal of Press/Politics. 2018;23(4):423–38. doi: 10.1177/1940161218790035 30886670 PMC6380726

[4] Alvares C, Dahlgren P. Populism, extremism and media: Mapping an uncertain terrain. European Journal of Communication. 2016;31(1):46–57. doi: 10.1177/0267323115614485

[5] Nabers D, Stengel FA. Sedimented practices and American identity in Donald J. Trump’s election campaign. In: Stengel FA, MacDonald DB, Nabers D, editors. Populism and world politics: Exploring inter- and transnational dimensions. Cham, Switserland: Springer International Publishing; 2019. p. 103–35.

[6] Kroes R. Signs of fascism rising. Society. 2017;54(3):218–25. doi: 10.1007/s12115-017-0128-7 28713183 PMC5491682

[7] Cramer KJ. The politics of resentment: Rural consciousness in Wisconsin and the rise of Scott Walker. Chicago, IL: The University of Chicago Press; 2016.

[8] Blassnig S, Ernst N, Büchel F, Engesser S, Esser F. Populism in online election coverage. Journalism Studies. 2018:1–20. doi: 10.1080/1461670X.2018.1487802

[9] Mudde C, Rovira Kaltwasser C. Studying populism in comparative perspective: Reflections on the contemporary and future research agenda. Comparative Political Studies. 2018;51(13):1667–93. doi: 10.1177/0010414018789490

[10] Hameleers M, Bos L, de Vreese CH. The appeal of media populism: The media preferences of citizens with populist attitudes. Mass Communication and Society. 2017;20(4):481–504. doi: 10.1080/15205436.2017.1291817

[11] Hameleers M, Bos L, de Vreese CH. Selective exposure to populist communication: How attitudinal congruence drives the effects of populist attributions of blame. Journal of Communication. 2018;68(1):51–74. doi: 10.1093/joc/jqx001

[12] Bos L, van der Brug W, de Vreese CH. An experimental test of the impact of style and rhetoric on the perception of right-wing populist and mainstream party leaders. Acta Politica. 2013;48(2):192–208. doi: 10.1057/ap.2012.27

[13] Hameleers M, Schmuck D. It’s us against them: a comparative experiment on the effects of populist messages communicated via social media. Information, Communication & Society. 2017;20(9):1425–44. doi: 10.1080/1369118X.2017.1328523

[14] Müller P, Schemer C, Wettstein M, Schulz A, Wirz DS, Engesser S, et al. The polarizing impact of news coverage on populist attitudes in the public: Evidence from a panel study in four European democracies. Journal of Communication. 2017;67(6):968–92. doi: 10.1111/jcom.12337

[15] Eveland WP, Coduto KD, Appiah O, Bullock OM. Listening during political conversations: Traits and situations. Political Communication. 2020;37(5):656–77. doi: 10.1080/10584609.2020.1736701

[16] Mudde C. Populism: An ideational approach. In: Kaltwasser CR, Taggart P, Espejo PO, Ostiguy P, editors. The Oxford handbook of populism: Oxford University Press; 2017. p. 609–12.

[17] Mudde C, Rovira Kaltwasser C. Populism. In: Freeden M, Stears M, editors. The Oxford handbook of political ideologies: Oxford University Press; 2013. p. 1–23.

[18] Jagers J, Walgrave S. Populism as political communication style: An empirical study of political parties’ discourse in Belgium. European Journal of Political Research. 2007;46(3):319–45. doi: 10.1111/j.1475-6765.2006.00690.x

[19] Hawkins KA, Rovira Kaltwasser C. Measuring populist discourse in the United States and beyond. Nature Human Behaviour. 2018;2(4):241–2. doi: 10.1038/s41562-018-0339-y 30936543

[20] Sorensen L. Populist communication in the new media environment: A cross-regional comparative perspective. Palgrave Communications. 2018;4:1–12. doi: 10.1057/s41599-018-0101-0

[21] Mazzoleni G. Populism and the media. In: Albertazzi D, McDonnell D, editors. Twenty-first century populism: The spectre of Western European democracy. London: Palgrave Macmillan UK; 2008. p. 49–64.

[22] Valkenburg PM, Peter J. The differential susceptibility to media effects model. Journal of Communication. 2013;63(2):221–43. doi: 10.1111/jcom.12024

[23] Tajfel H, Turner JC. An integrative theory of intergroup conflict. In: Austin WG, Worchel S, editors. The social psychology of intergroup relations. Monterey, CA: Brooks/Cole; 1979. p. 33–47.

[24] Stier S, Kirkizh N, Froio C, Schroeder R. Populist attitudes and selective exposure to online news: A cross-country analysis combining web tracking and surveys. The International Journal of Press/Politics. 2020;25(3):426–46. doi: 10.1177/1940161220907018

[25] Skovsgaard M, Andersen K. Conceptualizing news avoidance: Towards a shared understanding of different causes and potential solutions. Journalism Studies. 2020;21(4):459–76. doi: 10.1080/1461670X.2019.1686410

[26] Herne K, Christensen HS, Grönlund K. The influence of political knowledge on opinion polarization in citizen deliberation. Political Research Exchange. 2019;1(1):1–23. doi: 10.1080/2474736X.2019.1702887

[27] Stanyer J, Salgado S, Strömbäck J. Populist actors as communicators or politcal actors as populist communicators: Cross-national findings and perspectives. In: Aalberg T, Esser F, Reinemann C, Strömbäck J, de Vreese CH, editors. Populist political communication in Europe. New York, NY: Routledge; 2017. p. 353–64.

[28] Matthes J, Schmuck D. The effects of anti-immigrant right-wing populist ads on implicit and explicit attitudes: A moderated mediation model. Communication Research. 2017;44(4):556–81. doi: 10.1177/0093650215577859

[29] Schulz A. Where populist citizens get the news: An investigation of news audience polarization along populist attitudes in 11 countries. Communication Monographs. 2018;0(0):1–24. doi: 10.1080/03637751.2018.1508876

[30] Reinemann C, Scherr S, Stanyer J, Aalberg T, van Aelst P, Berganza R, et al. Cross-conceptual architecture of news. In: De Vreese CH, Esser F, Hopmann DN, editors. Comparing political journalism. London, England: Routledge; 2017. p. 150–67.

[31] Krämer B. Media populism: A conceptual clarification and some theses on its effects. Commun Theory. 2014;24(1):42–60. doi: 10.1111/comt.12029

[32] Westerwick A, Johnson BK, Knobloch-Westerwick S. Change Your Ways: Fostering Health Attitudes Toward Change Through Selective Exposure to Online Health Messages. Health Communication. 2017;32(5):639–49. doi: 10.1080/10410236.2016.1160319 27367925

[33] Peer E, Vosgerau J, Acquisti A. Reputation as a sufficient condition for data quality on Amazon Mechanical Turk. Behavior Research Methods. 2014;46(4):1023–31. doi: 10.3758/s13428-013-0434-y 24356996

[34] Leiner DJ, Scherr S, Bartsch A. Using open-source tools to measure online selective exposure in naturalistic settings. Communication Methods and Measures. 2016;10(4):199–216. doi: 10.1080/19312458.2016.1224825

[35] Wells GL, Windschitl PD. Stimulus sampling and social psychological experimentation. Personality and Social Psychology Bulletin. 1999;25(9):1115–25. doi: 10.1177/01461672992512005

[36] Buss AH, Perry M. The aggression questionnaire. Journal of Personality and Social Psychology. 1992;63(3):452–9. doi: 10.1037//0022-3514.63.3.452 1403624

[37] Lucas T, Zhdanova L, Alexander S. Procedural and distributive justice beliefs for self and others: Assessment of a four-factor individual differences model. Journal of Individual Differences. 2011;32(1):14–25. doi: 10.1027/1614-0001/a000032 WOS:000286379700002.

[38] Akkerman A, Mudde C, Zaslove A. How populist are the people? Measuring populist attitudes in voters. Comparative Political Studies. 2014;47(9):1324–53. doi: 10.1177/0010414013512600

[39] Kunda Z. The case for motivated reasoning. Psychological Bulletin. 1990;108(3):480–98. doi: 10.1037/0033-2909.108.3.480 2270237

[40] Clifford S, Jewell RM, Waggoner PD. Are samples drawn from Mechanical Turk valid for research on political ideology? Research & Politics. 2015;2(4):2053168015622072. doi: 10.1177/2053168015622072

[41] Thomas KA, Clifford S. Validity and Mechanical Turk: An assessment of exclusion methods and interactive experiments. Computers in Human Behavior. 2017;77:184–97. doi: 10.1016/j.chb.2017.08.038

[42] Levay KE, Freese J, Druckman JN. The demographic and political composition of mechanical turk samples. SAGE Open. 2016;6(1):2158244016636433. doi: 10.1177/2158244016636433

